# Production of shikimic acid from *Escherichia coli* through chemically inducible chromosomal evolution and cofactor metabolic engineering

**DOI:** 10.1186/1475-2859-13-21

**Published:** 2014-02-10

**Authors:** Yan-Yan Cui, Chen Ling, Yuan-Yuan Zhang, Jian Huang, Jian-Zhong Liu

**Affiliations:** 1 Biotechnology Research Center and Biomedical Center, School of Life Sciences, Sun Yat-sen University, Guangzhou 510275, P R China; 2 Current address: School of Materials Science and Engineering, South China University of Technology, Guangzhou 510641, P R China

**Keywords:** Shikimic acid, *Escherichia coli*, Chemically induced chromosomal evolution, NADPH, Transhydrogenase, NAD kinase

## Abstract

**Background:**

Shikimic acid (SA) produced from the seeds of Chinese star anise (*Illicium verum*) is a key intermediate for the synthesis of neuraminidase inhibitors such as oseltamivir (Tamiflu®), an anti-influenza drug. However, plants cannot deliver a stable supply of SA. To avoid the resulting shortages and price fluctuations, a stable source of affordable SA is required. Although recent achievements in metabolic engineering of *Escherichia coli* strains have significantly increased SA productivity, commonly-used plasmid-based expression systems are prone to genetic instability and require constant selective pressure to ensure plasmid maintenance. Cofactors also play an important role in the biosynthesis of different fermentation products. In this study, we first constructed an *E. coli* SA production strain that carries no plasmid or antibiotic marker. We then investigated the effect of endogenous NADPH availability on SA production.

**Results:**

The *pps* and *csrB* genes were first overexpressed by replacing their native promoter and integrating an additional copy of the genes in a double gene knockout (*aroK* and *aroL*) of *E. coli*. The *aroG*^*fbr*^, *aroB*, *aroE* and *tktA* gene cluster was integrated into the above *E. coli* chromosome by direct transformation. The gene copy number was then evolved to the desired value by triclosan induction. The resulting strain, *E. coli* SA110, produced 8.9-fold more SA than did the parental strain *E. coli* (Δ*aroK*Δ*aroL*). Following qRT-PCR analysis, another copy of the *tktA* gene under the control of the 5P_tac_ promoter was inserted into the chromosome of *E. coli* SA110 to obtain the more productive strain *E. coli* SA110. Next, the NADPH availability was increased by overexpressing the *pntAB* or *nadK* genes, which further enhanced SA production. The final strain, *E. coli* SA116, produced 3.12 g/L of SA with a yield on glucose substrate of 0.33 mol/mol.

**Conclusion:**

An SA-producing *E. coli* strain that carries neither a plasmid nor an antibiotic marker was constructed by triclosan-induced chromosomal evolution. We present the first demonstration that increasing NADPH availability by overexpressing the *pntAB* or *nadK* genes significantly enhances SA production.

## Background

Shikimic acid is a key intermediate for the synthesis of the neuraminidase inhibitor oseltamivir (Tamiflu®), an anti-influenza treatment [1]. The main commercial source of SA is seeds of the *Illicium* plant, such as *I. verum* or *I. anistatum*. However, the conventional method of producing SA from *I. verum* is typically low-yield and costly. Therefore, researchers have developed several metabolic engineering approaches to overproduce SA in *E. coli*[2,3]. These approaches are based on genetic modifications of central carbon metabolism and SA pathways. Specifically, the aromatic amino acid pathway is blocked after the SA production stage by deleting the *aroK* and *aroL* genes encoding shikimate kinase I and II. To increase the carbon flux from central carbon metabolism entering the aromatic amino acid pathway, researchers have amplified the feedback resistant 3-deoxy-D-*arabino*heptulosonate 7-phosphate (DAHP) synthase genes *aroF*^*fbr*^ or *aroG*^*fbr*^[4-8]. These modifications are commonly complemented with over-expression of the *aroB* and *aroE* genes [4-8]. Over-expression of the *tktA* gene (encoding transketolase) enhances the availability of erythrose 4-phosphate (E4P), and consequently increases the SA titer from 38 g/L to 52 g/L [6]. Over-expression of the *pps* gene (encoding phosphoenolpyruvate (PEP) synthase) has elevated the SA titer to 66 g/L [7]. SA production can also be increased by inactivating the phosphoenolpyruvate:carbohydrate phosphotransferase system (PTS) operon, combined with expressing the ATP dependent uptake and phosphorylation system comprising the glucose facilitator and the glucokinase from *Zymomonas mobili*[7,8]. *E. coli* engineered in this way yielded SA concentrations as high as 87 g/L in minimum medium supplemented with yeast extract [7]. The constitutive and synchronous expression of a six-gene synthetic operon (*aroG*^*fbr*^, *aroB*, *aroD*, *aroE*, *tktA* and *zwf*), in a laboratory-evolved strain bearing simultaneous PTS and *pykF* inactivations, was recently reported to increase the SA yield on glucose to 42% mol/mol, which represents the highest reported yield [9].

However, all of the above studies use plasmids for gene expression. Among the drawbacks of plasmid-based expression systems are structural and segregational instability, and allele segregation [10-12]. These plasmid instabilities cause genetic instability, with decreased productivity of the desired compound. Tyo et al. [13] reported that plasmid-carrying strains lose poly-3-hydroxybutyrate productivity after 40 rounds of subculturing. Moreover, the markers used for selecting and maintaining plasmids in hosts during cultivation are usually antibiotic resistance genes. However, antibiotics are both costly and banned from food and pharmaceutical production processes. In addition, the potential spread of antibiotic-resistant marker to natural microbes requires serious consideration. The likely outcome of this scenario is rapid emergence of multidrug-resistant organisms (e.g., superbacteria) [14,15]. To overcome these drawbacks of plasmid constructs, Tyo et al. [13] developed a plasmid-free method that achieves high copy numbers of the desired genes, termed chemically induced chromosomal evolution (CIChE). However, the λInCh genomic integration protocol used in CIChE is complicated and time-consuming, and Tyo et al.’s CIChE strains still carry an antibiotic resistance marker (chloramphenicol resistance) [13]. In our previous paper [16], we reported a modified CIChE that overcomes the drawbacks of Tyo et al’s original CIChE protocol. The resulting strain, developed by triclosan-induced chromosomal evolution, carries neither a plasmid nor an antibiotic marker. Thus, our first task in this study was to construct an SA-producing *E. coli* strain by triclosan-induced chromosomal evolution.

Cofactors are also known to play an important role in the biosynthesis of different fermentation products. Once the enzyme levels are no longer limiting, cofactor availability can become limiting and productivity must be boosted by cofactor manipulation. *In silico* flux analysis has identified a potentially important role for intracellular NADPH concentration in SA biosynthesis [17]. Thus, we also investigated the effect of endogenous NADPH availability on SA production by over-expressing the genes involved in NADPH synthesis.

## Results and discussion

### Inactivation of genes encoding shikimate kinase I and II, and chromosomal promoter replacement

To construct an SA-accumulating host strain, we initially focused on preventing the conversion of SA to chorismic acid in *E. coli*. To this end, we deleted the *aroK* and *aroL* genes to obtain *E. coli* BW25113 (*ΔaroKΔaroL*). PEP synthase (encoded by the *pps* gene) converts pyruvic acid to PEP. Carbon storage regulator CsrA controls glycogen synthesis and modulates the levels of three enzymes that directly participate in PEP metabolism: pyruvate kinase, PEP carboxykinase and PEP synthase (which synthesize PEP from oxaloacetate and pyruvate, respectively). The first of these is positively regulated, while both PEP enzymes are negatively regulated, by CsrA [18,19]. Because CsrA activity is antagonized by CsrB [18], PEP availability was increased by replacing the native promoters of the *pps* and *csrB* genes with the *P*_*lacQ1*_ promoter in *E. coli* BW25113 (*ΔaroKΔaroL*), yielding *E. coli* BW25113 (*ΔaroKΔaroL*, *P*_*pps*_*::P*_*lacQ1*_*, P*_*csrB*_*::P*_*lacQ1*_). SA production in this strain was 53.3% higher than in *E. coli* BW25113 (*ΔaroKΔaroL*) ((0.23 ± 0.02) g/L vs (0.15 ± 0.04) g/L; *P <* 0.01; Table 1). To further improve PEP availability, an additional copy of the *pps* and *csrB* genes controlled by the T5 promoter was inserted into *E. coli* BW25113 (*ΔaroKΔaroL*, *P*_*pps*_*::P*_*lacQ1*_*, P*_*csrB*_*::P*_*lacQ1*_) to obtain *E. coli* BW25113 (*ΔaroKΔaroL*, *P*_*pps*_*::P*_*lacQ1*_*, P*_*csrB*_*::P*_*lacQ1*_*, P*_*T5*_*-pps, P*_*T5*_*-csrB*). SA production in this strain was 73.3% higher than in *E. coli* BW25113 (*ΔaroKΔaroL*) ((0.26 ± 0.02) g/L vs (0.15 ± 0.04) g/L; *P <* 0.01; Table 1). These modifications also resulted in an increment in yield (mol SA/mol glucose) of about 80%. Over-expressing the *pps* gene increased DAHP production almost twofold, to approach the theoretical maximum [20]. DAHP is the precursor of SA and the condensation product of PEP and E4P, mediated by DAHP synthase. They thought that over-expressing the *pps* gene drives pyruvate to be recycled back to PEP [20]. Yakandawala et al. [18] achieved an approximately twofold increase in phenylalanine production by over-expressing the *csrB* gene. SA is the precursor of phenylalanine. Chandran et al. reported that over-expressing the *pps* gene raised the SA titer of 26% (from 52 to 66 g/L) [7]. Thus, the increments in SA titer and yield could be the consequence of overexpression of both *pps* and *csrB* genes.

**Table 1:** Shikimic acid (SA) production by different strains

**Stain**	**OD**_**600**_	**SA concentration (g/L)**	**Yield (mol SA/mol glucose)**
BW25113 (Δ*aroK*Δ*aroL*)	7.18 ± 0.95	0.15 ± 0.04	0.05
BW25113 (Δ*aroK*Δ*aroL*, *P*_*pps*_*::P*_*lacQ1*_*, P*_*csrB*_*::P*_*lacQ1*_)	5.82 ± 0.04	0.23 ± 0.02	0.08
BW25113 (Δ*aroK*ΔaroL, *P*_*pps*_*::P*_*lacQ1*_*, P*_*csrB*_*::P*_*lacQ1*_*, P*_*T5*_*-pps, P*_*T5*_*-csrB*)	5.82 ± 0.57	0.26 ± 0.02	0.09
SA110	5.91 ± 1.34	1.34 ± 0.15	0.21
SA112	5.85 ± 0.46	1.70 ± 0.01	0.25

**Figure 1:**
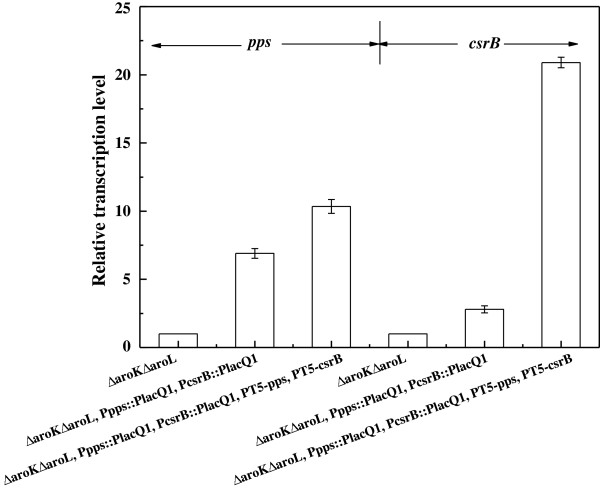
**Transcription levels of the *pps *** **and *csrB *** **genes in the different strains.**

Quantitative real-time PCR analysis demonstrates that the above modifications indeed enhanced the transcription levels of the *pps* and *csrB* genes (Figure 1). The replacement of the native promoter resulted in the upregulations of the transcription levels of the *pps* and *csrB* genes about 6.9- and 2.8-fold (*P <* 0.01), respectively. Integrating an additional copy of the two genes further upregulated the transcription levels of the *pps* and *csrB* genes about 10.4- and 20.9-fold (*P <* 0.01), respectively.

### Chemically induced chromosomal evolution

**Figure 2:**
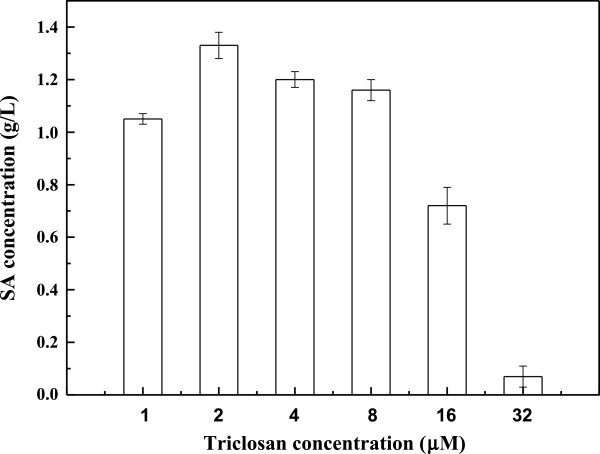
Shikimic acid production of CIChE strains at different triclosan concentrations.

**Figure 3:**
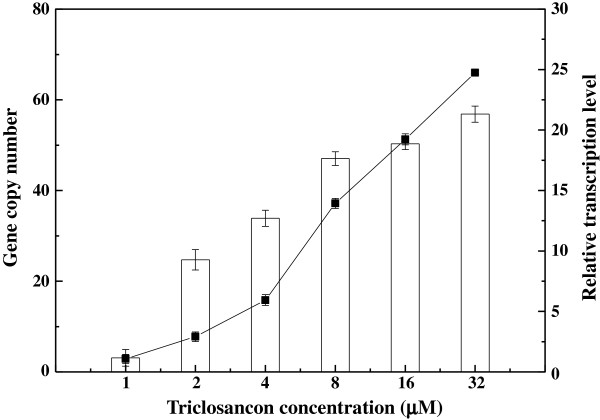
**Gene copy number (line) and transcription level (column) of the *aroE *** **gene in CIChE strains.**

Many papers reported that plasmid-based over-expression of feedback-resistant DAHP synthases (coded by *aroF*^*fbr*^ or *aroG*^*fbr*^), shikimate dehydrogenase (coded by *aroE*), transketolase (ecoded by *tktA*), and DHQ synthase enzymes (ecoded by *aroB*) [2-9]. To overcome the drawbacks of plasmid expression systems, the *aroG*^*fbr*^, *tktA, aroB* and *aroE* gene cluster was inserted into the chromosome of *E. coli* BW25113 (*ΔaroKΔaroL*, *P*_*pps*_*::P*_*lacQ1*_*, P*_*csrB*_*::P*_*lacQ1*_*, P*_*T5*_*-pps, P*_*T5*_*-csrB*) by direct transformation. The transformed strain was evolved to higher gene copy number by exposure to increasing triclosan concentrations. Figure 2 plots the SA production in CIChE strains resistant to different triclosan concentrations. The maximum SA concentration (1.34 ± 0.15) g/L was obtained by the CIChE strains resistant to 2 μM triclosan. When the triclosan concentration exceeded 2 μM, SA production of the CIChE strains did not further increase. Thus, the *recA* gene of the CIChE strain resistant to 2 μM triclosan was deleted to obtain *E. coli* SA110. Homologous recombination, which can potentially reduce the copy number, is inhibited in this strain. Figure 3 shows that the copy numbers and transcription levels of the *aroE* gene of the CIChE strains increase with triclosan concentration during chromosomal evolution. At a triclosan concentration of 2 μM, the copy number reached about 8 in the CIChE strain. When the triclosan concentration was above 2 μM, the gene copy numbers and transcription levels still increased; however, the SA production of the CIChE strains did not increase. The transcription levels of the other three genes show the similar trend (Additional file 1: Figure S1). The results indicated that there is an optimal copy number and transcription level of the gene cluster for efficient production of SA.

**Figure 4:**
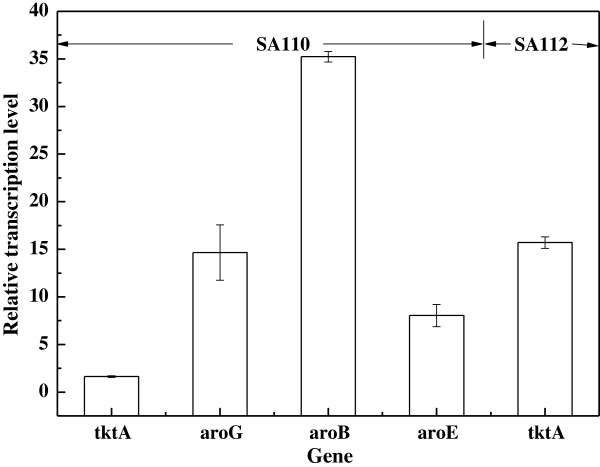
**Transcription levels of genes of interest in the CIChE strain *E. coli *SA110 and SA112 compared with those in *E. coli *BW25113 (**Δ***aroK***Δ***aroL*).**

We also analyzed the transcription levels of the *E. coli* SA110 genes by qRT-PCR, and compared the data with those of *E. coli* BW25113 (*ΔaroKΔaroL*) (Figure 4). The transcription levels of the *tktA, aroG*^*fbr*^*, aroB* and *aroE* genes in *E. coli* SA110 were about 1.6, 14.7, 35.2 and 8.1 times higher (*p* < 0.05) than in *E. coli* BW25113 (*ΔaroKΔaroL*). The transcription level of the *tktA* gene was the lowest. It may be because the distance of the *tktA* gene from the promoter is the longest [21]. They thought that the abundance of mRNA decreased monotonically with the increasing distance of the gene from the promoter in *E. coli*[21]*.* The gene ranked in front will be translated primarily, followed by the translation of the subsequent genes along with the mRNA transcription for polycistronic operons in *E. coli*. However, the transcription level of the second gene (the *aroB* gene) from the promoter was the highest. This could be the consequence of codon usage biases.

Because the *tktA* gene was least upregulated among these genes, we examined whether *tktA* is the bottleneck for SA production in *E. coli* SA110. An additional copy of the *tktA* gene under the control of the 5P_tac_ promoter was inserted into the chromosome of *E. coli* SA110 to obtain *E. coli* SA112. This modification raised the SA titer to (1.70 ± 0.01) g/L and the SA yield to 0.25 mol/mol (Table 1). Figure 4 shows that this integration upregurated the transcription level of the *tktA* gene. The transcription level of the *tktA* gene in *E. coli* SA112 was much higher than that in *E. coli* SA110. This may be because the additional integrated *tktA* gene was directly controlled by the stronger 5P_tac_ promoter.

### Effect of NADPH availability

To investigate the effect of NADPH availability on SA production, transhydrogenase (encoded by *sthA* or *pntAB*) and NAD kinase (encoded by *nadK*) were first amplified by plasmid-based over-expression in *E. coli* SA112. The results are presented in Table 2. Overexpression of the *pntAB* or *nadK* genes increased both SA production and the intracellular NADPH concentration. *E. coli* generates NADPH in one of three ways: the pentose phosphate (PP) pathway, isocitrate dehydrogenase and transhydrogenase. Two isoforms of transhydrogenase exist in *E. coli*, membrane-bound transhydrogenase (PntAB) and soluble transhydrogenase (SthA). During aerobic batch growth with glucose, 35–45% of the NADPH required for biosynthesis is produced via PntAB, while the PP pathway and isocitrate dehydrogenase generate 35–45% and 20–25%, respectively [22]. ATP-dependent NAD kinase encoded by *nadK* catalyzes the phosphorylation of NAD to NADP. Overexpression of *nadK* increases the size of the NADP pool, which potentially increases the abundance of NADPH. It can be seen in Table 2 that overexpression of the *sthA* gene increased SA titer and reduced the intracellular NADPH concentration. Moreover, the SA titer and intracellular NADPH concentration of the strain overexpressing *sthA* was lower than in strains overexpressing the *pntAB* or *nadK* genes. PntAB catalyzes the transfer of reducing power from NADH to NADP^+^ in an energy-dependent manner at low intracellular NADPH levels, while SthA mainly catalyzes the reoxidation of NADPH in an energy-independent manner when NADPH is abundant [22]. Thus, if the *pntAB* or *sthA* gene is overexpressed, the intracellular NADPH concentration is increased or decreased, respectively. As shown in Table 3, overexpressing the *sthA* gene indeed caused a reduction in intracellular NADPH concentration, with consequent reduction of the SA titer.

**Table 2:** **Effect of over-expressing genes involved in NADPH synthesis on SA production in *E. coli *** **SA112**

**Strain**	**OD**_**600**_	**SA concentration (g/L)**	**Yield (mol SA/mol glucose)**	**NADPH (μM)**
SA112 (pMP5)	4.79 ± 0.64	1.34 ± 0.09	0.22	1.93 ± 0.01
SA112 (pMPsthA)	5.78 ± 0.26	1.42 ± 0.07	0.24	1.61 ± 0.01
SA112 (pMPpntAB)	5.50 ± 0.11	1.89 ± 0.01	0.27	2.23 ± 0.01
SA112 (pMPnadK)	5.20 ± 0.34	1.92 ± 0.01	0.29	2.55 ± 0.01
SA114	6.55 ± 0.30	2.99 ± 0.01	0.31	4.51 ± 0.01
SA116	6.40 ± 0.51	3.12 ± 0.01	0.33	5.77 ± 0.01

**Table 3:** **Effect of overexpressing the *sthA *** **gene on SA production in *E. coli *** **SA116**

**Strain**	**OD**_**600**_	**SA concentration (g/L)**	**Yield (mol SA/mol glucose)**	**NADPH (μM)**
SA116(pMP5)	5.56 ± 0.76	1.83 ± 0.07	0.23	1.67 ± 0.02
SA116(pMPsthA)	6.73 ± 0.11	1.69 ± 0.11	0.20	0.74 ± 0.04

**Figure 5:**
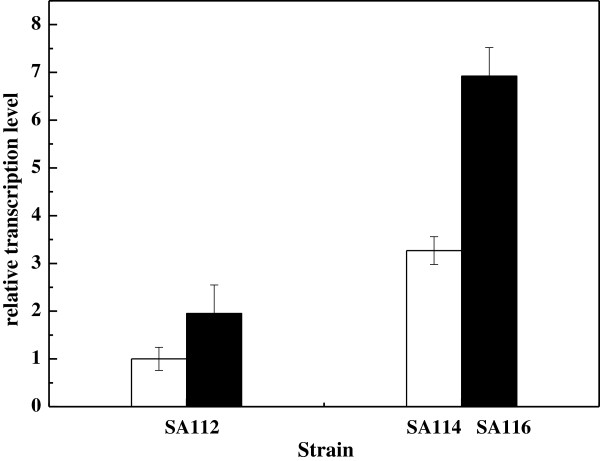
**Transcription levels of the *pntAB *** **(open bar) and *nadK *** **(black bar) in the different strains.**

Because plasmid-based overexpression of *sthA* showed lower the SA titer compared to overexpression of the *pntAB* or *nadK* genes, to alleviate the metabolic burden caused by the plasmid, the *pntAB* and *nadK* genes were integrated into the chromosome of *E. coli* SA112 to obtain *E. coli* SA114 and SA116, respectively. Chromosomal overexpression enhanced the intracellular NADPH concentration and SA titer relative to plasmid overexpression (Table 2), and also improved the cell growth. It can be seen in Figure 5 that the transcription levels of the *pntAB* and *nadK* genes were enhanced about 3.3- and 3.6-fold (*P <* 0.01) after chromosomal integration, respectively.

This study demonstrated a strong correlation between NADPH availability and SA production. In the SA biosynthesis pathway, shikimate dehydrogenase (encoded by the *aroE* gene) catalyzes the reduction of 3-dehydroshikimate to shikimate, which requires NADPH as a cofactor. These CIChE strains (e.g. SA110, 112, 114, 116), in which the *aroE* gene was overexpressed and therefore required more NADPH for SA biosynthesis. Although NADPH can be provided by the oxidative part of the PP pathway, this branch is unfavorable for SA production. Stoichiometric analysis shows that the oxidative part of the PP pathway is not required in the reaction scheme. Glucose enters the nonoxidative part of the PP pathway, where it provides E4P for maximum theoretical SA yield [20]. Accordingly, the overall reaction for SA biosynthesis from glucose is: 7 glucose + 6 ATP + 6 NAD + 6 NADPH → 6 SA + 6 ADP + 6 NADH + 6 NADH + 6 NADP + 12H^+^ + 6 HPO_4_^2+^. Increased intracellular NADPH via overexpression of the *pntAB* or *nadK* genes favored SA production (Table 2), suggesting that more carbon flux was channeled into the nonoxidative part of the PP pathway for E4P biosynthesis. *In silico* flux analysis has demonstrated a potentially important role for intracellular NADPH concentration in SA biosynthesis. Under different genetic and environmental conditions, *E. coli* cells consume the same quantities of NADPH to maximize their SA production [17]. According to some studies, maintaining NADPH availability improves metabolite production. For instance, overexpressing *E. coli pntAB* in *Corynebacterium glutamicum* enhances NADPH availability, in turn increasing the intracellular levels of L-lysine [23] and L-ornithine [24]. Overexpression of *nadk* in *E. coli* increases the NADPH/NADP ratio, thereby enhancing thymidine biosynthesis [25]*.* In *C. glutamicum*, overexpressing the NAD kinase gene improves L-lysine [26] and L-ornithine [24] production. Simultaneous chromosomal overexpression of transhydrogenase (*pntAB*) and NAD kinase (*yfjB*) genes in *E. coli* increases the NADPH supply and improves anaerobic isobutanol production [27].

The SA yield obtained in the present study is below the highest value (0.42 mol/mol) reported in the literature [9], achieved by the constitutive and synchronous expression of a six-gene synthetic operon, in a laboratory-evolved strain bearing simultaneous PTS and *pykF* inactivations in a 1 L bioreactor containing 100 g/L of glucose and 30 g/L of yeast extract. However, the SA yield obtained in this research is on part with the 33% yield reported by Chandran et al. [7]. They reported the highest SA titer (87 g/L). It remains far from the theoretical maximum (86% mol/mol) [20], implying a need for further research to improve this value. Currently, we are exploring whether inactivation of PTS, *pykF* and the oxidative part of the PP pathway ensures a high SA yield. The results of these modifications will be reported in a subsequent paper.

## Conclusion

To overcome the drawbacks of plasmid-based expression systems, the *aroG*^*fbr*^, *aroB*, *aroE* and *tktA* gene cluster was integrated into the *E. coli* chromosome by direct transformation. The gene copy number was then evolved to the desired value by triclosan induction. Following qRT-PCR analysis, SA production was further enhanced by inserting a second copy of the *tktA* gene under the control of the 5P_tac_ promoter into the chromosome of the CIChE strain. The effect of NADPH availability on SA production was also investigated. NADPH availability and SA production were found to be strongly correlated. Plasmid-based or chromosomal overexpression of the *pntAB* or *nadK* genes enhanced the intracellular NADPH concentration and consequently the SA titer. This is the first report of an engineered SA-producing strain of *E. coli* that lacks both a plasmid and an antibiotic marker. Using this strain, no resistance-conferring compound was required during the fermentation process.

## Methods

### Strains, plasmids and primers

The strains and plasmids used in this study are listed in Table 4. *E. coli* DH5α was used for plasmid construction. The parent strain for SA production was *E. coli* BW2511 [28]. The primers used in this study are listed in Table 5.

**Table 4:** Strains and plasmids used in this study

**Strain/plasmid**	**Description**	**Source or reference**
Strain		
*E. coli* DH5α	*supE44* Δ(*lacZYA-argF*) *U169* (Φ*80lacZ* Δ*M15*) *hsdR17 recA endA1 gyrA96 thi-1 relA1*	Invitrogen
*E. coli* BW25113	*lacI*^q^*rrnB*_T14_ Δ*lacZ*_WJ16_*hsdR514* Δ*araBAD*_AH33_ Δ*rhaBAD*_LD78_	28
*E. coli* BW25113 (Δ*aroK*Δ*aroL*)	*E. coli* BW25113, Δ*aroK*Δ*aroL*	This study
*E. coli* BW25113 (Δ*aroK*Δ*aroL*, *P*_*pps*_*::P*_*lacQ1*_*, P*_*csrB*_*::P*_*lacQ1*_)	*E. coli* BW25113, Δ*aroK*Δ*aroL*, replacement of the native promoter of the *pps* and *csrB* gene with the *P*_*lacQ1*_ promoter	This study
*E. coli* BW25113 (Δ*aroK*Δ*aroL*, *P*_*pps*_*::P*_*lacQ1*_*, P*_*csrB*_*::P*_*lacQ1*_*, P*_*T5*_*-pps, P*_*T5*_*-csrB*)	*E. coli* BW25113(Δ*aroK*Δ*aroL*, *P*_*pps*_*::P*_*lacQ1*_*, P*_*csrB*_*::P*_*lacQ1*_) with an additional chromosomal copy of the *pps* and *csrB* genes under the control of the T5 promoter	This study
SA110	CIChE strain of the *aroG*^*fbr*^*, tktA, aroB* and *aroE* gene cluster resistance to 2 μM triclosan from *E. coli* BW25113 (Δ*aroK*Δ*aroL*, *P*_*pps*_*::P*_*lacQ1*_*, P*_*csrB*_*::P*_*lacQ1*_), Δ*recA*	This study
SA112	SA110 with an additional chromosomal copy of the *tktA* gene under the control of the 5P_tac_ promoter (5 tandem repeats of the core-tac-promoter)	This study
SA114	SA112 with an additional chromosomal copy of the *pntAB* genes under the control of the 5P_tac_ promoter	This study
SA116	SA112 with an additional chromosomal copy of the *nadK* gene under the control of the 5P_tac_ promoter	This study
Plasmid		
pHKKF3T5b	CIChE integration expression vector, *attP*_HK_ site, Kan^r^	16
pHKKT5b	Integration expression plasmid, *attP*_HK_ site, P_T5_ promoter, Kan^r^	33,34
pP21KT5b	Integration expression plasmid, *attP*_P21_ site, P_T5_ promoter, Kan^r^	33,34
pAH69	Helper plasmid expressing phage HK022 Int, Amp^r^	31
pAH121	Helper plasmid expressing phage P21 Int, Amp^r^	31
pCP20	pSC101 replicon^ts^ Flp(λR*p*) *cI*857, Cm^r^, Amp^r^	28
pKD3	*oriRγ*, *FRT*::*cat*::*FRT* template plasmid, Cm^r^, Amp^r^	28
pSIM6	pSC101 replicon^ts^ P_L_*-gam-bet-exo cI*857, Amp^r^	29
p5TG	pSC101 replicon^ts^, 5P_tac_ promoter, Spc^r^	32
pMP5	P5TG derivative, pSC101 *ori*, Constitutive expression, Spc^r^	This study
pBAD24	pMB1 *ori*, P_BAD_ L-arabinose inducible, Amp^r^	30
pBAD-csrB-pps	pBAD24 derivative containing the *csrB* and *pps* genes	Lab storage
pBEB	pBAD24 derivative containing the *aroB* and *aroE* genes	This study
pBEBG	pBAD24 derivative containing the *aroG*^*fbr*^*, aroB* and *aroE* genes	This study
pHKEBG	pHKKF3T5b derivative containing *aroG*^*fbr*^*, aroB* and *aroE* genes	This study
pHKEBGT	pHKKF3T5b derivative containing *aroG*^*fbr*^*, tktA, aroB* and *aroE* genes	This study
pMPsthA	pMP5 derivative containing *sthA* gene	This study
pMPpntAB	pMP5 derivative containing *pntAB* genes	This study
pMPnadK	pMP5 derivative containing *nadK* gene	This study
pP21KT5b-csrB-pps	pP21KT5b derivative containing *csrB* and *pps* genes	This study
pHKKT5b-tktA	pHKKT5b derivative containing *tktA* gene	This study
pHKK5Tacb	Integration expression plasmid, *attP*_HK_ site, 5tac promoter, Kan^r^	This study
pP21K5Tacb	Integration expression plasmid, *attP*_P21_ site, 5tac promoter, Kan^r^	This study
pP21K5Tacb-pntAB	pHK5Tacb derivative containing *pntAB* genes	This study
pP21K5Tacb-nadk	pHK5Tacb derivative containing *nadK* gene	This study

**Table 5:** Primers used in this study

**Primer**	**Sequence and purpose**^**a**^
LP1	5’-ATGACACAACCTCTTTTTCTGATCGGGCCTCGGGGCTGTGGTAAAACAACAGCGATTGTGTAGGCTGGAG-3’, deletion of *aroL*
LP2	5’-TCAACAATTGATCGTCTGTGCCAGGGCGCTGCGAATTTCAGAAATCACCTTAACGGCTGACATGGGAATTAG-3’, deletion of *aroL*
P1	5’-GTTTCGTTGGCATCGTTCTT-3’, Diagnostic PCR for the deletion of *aroL*
P2	5’-ATTCTCATGACACCGGCTTT-3’, Diagnostic PCR for the deletion of *aroL*
KP1	5’-ATGGCAGAGAAACGCAATATCTTTCTGGTTGGGCCTATGGGTGCCGGAAAAGCGATTGTGTAGGCTGGAG-3’, deletion of *aroK*
KP2	5’-TTAGTTGCTTTCCAGCATGTGAATAATCTGGTTTGCAACCACTTTAGCGCTTAACGGCTGACATGGGAATTAG-3’, deletion of *aroL*
P3	5’-GCGAAGCGGGTTTATCATTA-3’, Diagnostic PCR for the deletion of *aroK*
P4	5’-GTTCCCCGAGAGTAACGAC-3’, Diagnostic PCR for the deletion of *aroK*
GP1	5*’*-GCCTGCAGAGGAGGGCGTAAATATGAATTATCAGAACGACGATT-3’, *Pst*I, PCR for *aroG*
GP2	5’-CGGCATGCTTACCCGCGACGCGCTTTTACT-3’, *Sph*I, PCR for *aroG*
G15P1	5’-CTCAATATGATCACCCCACAAT-3’, sited-specific mutagenesis of *aroG*
G15P2	5’-AAACTCACCTGCCGCTGGCAGACCG-3’, sited-specific mutagenesis of *aroG*
EP1	5’-GCGAATTCAGGAG*G*TAATAAATATGGAAACCTAT GCTGTTTT-3’, *Eco*RI, PCR for *aroE*
EP2	5’-CGGCGGCCGCTTA*T*CACGCGGACAATTCCTCCT-3’, *Not*I, PCR for *aroE*
BP1	5’-CGGCGGCCGCAGGAG*G*TAATAAATATGGAGAGGATTGTCGTTAC-3’, *Not*I, PCR for *aroB*
BP2	5’-GCCCATGGTTACGCTGATTGACAATCGG-3’, *Nco*I, PCR for *aroB*
TP1	5’-GCGCATGCAGGAG*G*TAATAAATATGTCCTCACG TAAAGAGCT-3’, *Sph*I, PCR for *tktA*
TP2	5’-CGGAGCTCTTACAGCAGTTCTTTTGCTTTCG-3’, *Sac*I, PCR for *tktA*
PntF	5’-CAGGGTACCTCATCAATAAAACCG-3’, *Kpn*I, PCR for *pntAB*
PntR	5’-CGTCTGCAGTTACAGAGCTTTCAG-3’, *Pst*I, PCR for *pntAB*
SthF	5’-TTTTGGTACCCAGGTAAGCCCTACCATGC-3’, *Kpn*I, PCR for *sthA*
SthR	5’-GGGCTGCAGGGCCATTTCGATAAAGTTTT-3’, *Pst*I, PCR for *sthA*
NadF	5’-GCGGGGTACCATGAATAATCATTTCAAGTG-3’, *Kpn*I, PCR for *nadK*
NAdR	5’-GCGGTCTAGATTAGAATAATTTTTTTGACCA-3’, *Xba*I, PCR for *nadK*
5TacF	5’-ACGCGTGTAAAACGACGGCCAGT-3’, *Mlu*I, PCR for the 5P_tac_ promoter
5TacR	5'-CCGCGCATGCGGATCCGAATTCATGCATCTAGTATTTCTCCTCTTTAATGGAT-3’, *Sph*I, PCR for the 5P_tac_ promoter
CsrF	5’-CGGAGCTCAGGAGGTAATAAATGAGTCAGACAACGAAGTGAACAT-3’, *Sac*I, PCR for the *csrB* and *pps* gene cluster
PpsR	5’-CGGGTACCTTATTTCTTCAGTTCAGCCAGG-3’, *Kpn*I, PCR for the *csrB* and *pps* gene cluster
QEF	5’-GGATCGCCGGAATATCACCAC-3’, qPCR for the *aroE* gene
QER	5’-ACTACTGCCACTCCTTTCCCT-3’, qPCR for the *aroE* gene
QBF	5’-AAC GAA ACC CTG GCT CCT CTG-3’, qPCR for the *aroB* gene
QBR	5’-AAGCGCCACCAGCGTAGTATC-3’, qPCR for the *aroB* gene
QGF	5’-CGTTGCTGAAGTGAAAGAAGGG-3’, qPCR for the *aroG* gene
QGR	5’-ACGTCAGCACAAACATCCATC-3’, qPCR for the *aroG* gene
QTF	5’-TTTCGCCTGGCCTGCTTCTTT-3’, qPCR for the *tktA* gene
QTR	5’-CGACGCTGAAATTGCCCTGAC-3’, qPCR for the *tktA* gene
QNF	5’-TGGAATCAACCGTGGCAACCT-3’, qPCR for the *nakK*gene
QNR	5’-TGGAATCAACCGTGGCAACCT-3’, qPCR for the *nakK*gene
QPF	5’-AGCCGGAGTACGAGTTCAGCA-3’, qPCR for the *pntAB* gene
QPR	5’-ATTGCGCTGGTATTCGGCTGG-3’, qPCR for the *pntAB* gene
QPPF	5’-GACATCTTCTCGCTGACCAAC-3’, qPCR for the *ppsA g*ene
QPPR	5’-TTACCGGTGTGGCCATCTTTC-3’, qPCR for the *ppsA* gene
QCSF	5’-CTGGATGAAGCGAAGAGGATG-3’ , qPCR for the *csrB* gene
QCSR	5’-ATTGCTTCCTGCTCACACCAC-3’, qPCR for the *csrB* gene
QCF	5’-TTGTCGGCGGTGGTGATGTC-3’, qPCR for the *cysG* gene
QCR	5’-ATGCGGTGAACTGTGGAATAAACG-3’, qPCR for the *cysG* gene

^a^Restriction enzyme sites are underlined.

### Gene knockout and integration

Gene knockouts and replacement of the native promoter were carried out by PCR product recombination [28] using the pSIM6 plasmid [29] expressing the lambda red recombination system and pKD3 [28] as the template for PCR. Gene knockouts were verified by colony PCR using appropriate primers (listed in Table 5).

### Chemically induced chromosomal evolution

The *aroE, aroB* and *aroG* genes were amplified from K12 genomic DNA using the corresponding primer pairs (Table 5) and cloned into pMD18-T to obtain pMD-aroE, pMD-aroB and pMD-aroG, respectively. The feedback-inhibition-resistant (fbr) *aroG* gene, which contained an Asp-146-Asn substitution, was obtained by site-directed mutagenesis using the MutanBEST kit (Takara, Dalian, China), following the manufacturer’s instructions. The *aroE* and *aroB* fragments were digested by their corresponding restriction enzymes and inserted into the *Eco*RI/*Nco*I sites of pBAD24 [30] to form pBEB. The *aroG*^*fbr*^ fragment was digested from pMD-aroG^fbr^ and inserted into the *Pst*I/*Sph*I sites of pBEB to obtain pBEBG. The *aroG*^*fbr*^*, aroB* and *aroE* gene cluster was excised from pBEBG using restriction enzymes *Eco*RI and *Sph*I. The resulting gene cluster was cloned into the *Eco*RI/*Sph*I sites of pHKKF3T5b [16] to obtain pHKEBG. The *tktA* gene was amplified from *E. coli* using the primers TP1 and TP2. The fragment was digested along with pHKEBG using *Sph*I and *Sac*I, and ligated together to form pHKEBGT. Assisted by a helper plasmid, pAH69, which expresses the phage integrase [31], the resulting integration vector pHKEBGT was inserted into the bacterial attachment (*attB*) site of *E. coli* by direct transformation, as described by Chen et al. [16] and Chiang et al. [14].

CIChE of the above construct was carried out by subculturing the resulting strains in 5 mL Super Optimal Broth (SOB) medium with increasing concentrations of triclosan in 15 mL culture tubes, as described by Chen et al. [16] and Tyo et al. [13]. The strains were grown to stationary phase in 1 μM triclosan. Fifty milliliters of the culture was subcultured into a new culture tube, in which the triclosan concentration was doubled from 1 to 2 μM and allowed to grow to stationary phase. The process was repeated until the desired concentration (as high as 32 μM) was reached. The *recA* gene of the CIChE strain was then deleted.

### Plasmid construction

The multiple cloning site (ATGCATGACGTCGGGCCCGCATGCCACGTGGAGCTCGGTACCATAAAACGAAAGGCTCAGTCGAAAGACTGGGCCTTTCGTTTTATCAATTGCTGCAGCCCGGGCTCGAGTCTAGAGTCGACCCGCGG) was synthesized by Takara Biotechnology. This fragment was digested along with p5TG [32] using *Nsi*I/*Sac*II, and ligated with p5GT to form pMP5. The *sthA*, *pntAB* and *nadK* genes were amplified from *E. coli* using the corresponding primer pairs (Table 5) and then inserted into the corresponding sites of pMP5 to form pMPsthA, pMPpntAB and pMPnadK, respectively.

### Chromosomal integration

The *pps* and *csrB* gene cluster was amplified from pBAD-csrB-pps using the primers CsrF and PpsR and then inserted into the *SacI*/*Kpn*I sites of pP21KT5b [33,34] to obtain pP21KT5b-csrB-pps.

To enhance the expression of the genes on the integrate vector, the T5 promoter of the integrate expression plasmid was replaced with the 5P_tac_ promoter (five tandem repeats of the core-tac-promoter) [32]. The 5P_tac_ promoter was amplified using primers 5TacF and 5TacR from p5TG [32]. The resulting fragment was digested along with pHKKT5b or pP21KT5b [33,34] using *Mlu*I and *Sph*I, and was ligated into these plasmids to form pHKK5Tacb or pP21K5Tacb, respectively. The *tktA* gene was excised from pHKEBGT by *Sph*I and *Sac*I and ligated into the *Sph*I*/Sac*I sites of pP21K5Tacb to obtain the integrate vector pP21K5Tacb-tktA. The *pntAB* and *nadK* genes were excised from pMPpntAB and pMPnadk by their corresponding restriction enzymes and then inserted into the corresponding sites of pP21K5Tacb to form the integrate vectors pP21K5Tacb-pntAB and pP21K5Tacb-nadK, respectively.

Assisted by a helper plasmid, pAH121, expressing the phage integrase [31], the above integrate vectors (pP21KT5b-csrB-pps, pP21K5Tacb-tktA, pP21K5Tacb-pntAB and pP21K5Tacb-nadK) were inserted into the bacterial attachment (*attB*) site of *E. coli* by direct transformation, as described by Chen et al. [16] and Chiang et al. [14].

### Quantitative real-time PCR (qRT-PCR)

Total RNA from *E. coli* cells grown for 60 h in shake flasks was isolated using an RNA extraction kit (Dongsheng Biotech, Guangzhou, China), following the manufacturer’s instructions. The first-strand cDNA was synthesized using an All-in-One™ First-Strand cDNA Synthesis Kit (GeneCopoeia, Guangzhou, China). The qRT-PCR was performed with the All-in-One™ qPCR Mix kit (GeneCopoeia) on an iCycler iQ5 Real Time PCR system (Bio-Rad Laboratories, California, USA). The template was 100 ng of cDNA. The PCR conditions were: 95°C for 10 min, followed by 45 cycles of denaturation at 95°C for 10 s, annealing at 60°C for 20 s, and extension at 72°C for 15 s. The primers for qRT-PCR are presented in Table 4. Data were analyzed by the 2^-ΔΔCt^ method described by Livak and Schmittgen [35], and normalized by *cysG* gene expression.

Gene copy numbers were measured by qPCR on genomic DNA isolated from the appropriate CIChE strains. qPCR was performed as above. The primers QEF and QER (Table 5) were used to measure the copy number of *aroE*.

### SA production

For SA production, *E. coli* cells were precultured overnight in a falcon tube containing 5 mL Luria Broth (LB) at 37°C. The main cultures were incubated in the fermentation medium (pH 7.0) containing (g/L): glucose 10, peptone 1, proline 1.24, KH_2_PO_4_ 3, Na_2_HPO_4_ · 7H_2_O 13, MgCl_2_·6H_2_O 0.24, NaCl 0.5, NH_4_Cl 1, CaCl_2_ 0.1. The main cultures were inoculated with a starting OD_600_ of 0.1 and incubated at 37°C for 72 h in a rotary shaking incubator at 150 rpm.

### Assay

Cell growth was measured by optical density at 600 nm and converted to dry cell weight (DCW; g/L) using a standard curve. SA concentration was determined by HPLC using a Shimadzu system (LC-20A, Shimadzu, Japan) equipped with an Inertsil ODS-SP column (5 μm, 4.6 × 150 mm, GL Sciences Inc, Tokyo, Japan). The mobile phase was 5 mmol/L H_2_SO_4_, with a flow rate of 0.5 mL/min, at 50°C. SA was detected by a photodiode array detector (SPD-M20A) operating at 210 nm, and quantified by a standard curve constructed from serial dilutions of a SA standard stock solution (1 mg/ml, J&K Scientific Ltd, Beijing, China). Glucose concentration was determined by glucose oxidase using a glucose assay kit (Shanghai Rongsheng Biotech Corporation, Shanghai, China).

### NADPH assay

Following aerobic cultivation of *E. coli* on a rotary shaker (150 rpm) at 37°C for 54 h, the cells were harvested by centrifugation and washed twice with water. Intracellular NADPH was extracted and quantified using the Enzychrom™ NADP+/NADPH Assay kit (BioAssay Systems, Hayward, CA), following the manufacturer’s instructions.

### Statistical analysis

All experiments were conducted in triplicate, and data were averaged and presented as the mean ± standard deviation. Significant differences were determined by one-way analysis of variance followed by Tukey’s test, using the OriginPro (version 7.5) package. Statistical significance was defined as *p* < 0.05.

## Competing interests

The authors declare that they have no competing interests.

## Authors’ contributions

YYC carried out most of the experiments. CL amplified some of the genes. YYZ constructed some of the plasmids. JH deleted the *aroK* and *aroL* genes. JZL developed the concept and designed the method, led the project and drafted the manuscript. All authors read and approved the final manuscript.

## Supplementary Material

Additional file 1: Figure S1 The transcription levels of the *aroB*, *aroG*^*fbr*^ and *tktA* genes in CIChE strains. Click here for file
