# Supplementary material for: Production of shikimic acid from Escherichia coli through chemically inducible chromosomal evolution and cofactor metabolic engineering
Source: Microb Cell Fact. 2014 Feb 10;13:21. doi: 10.1186/1475-2859-13-21 (PMC3923554; doi:10.1186/1475-2859-13-21)
Supplement: Additional file 1: Figure S1 — The transcription levels of the aroB, aroG fbr and tktA genes in CIChE strains. [file 1475-2859-13-21-S1.docx]

Supplementary Figure for:

Production of shikimic acid from *E. coli* through chemically inducible chromosomal evolution and cofactor metabolic engineering

*Yan-Yan Cui, Chen Ling^a^,Yuan-Yuan Zhang, Jian Huang, Jian-Zhong Liu**

*Biotechnology Research Center and Biomedical Center, School of Life Sciences, Sun Yat-sen University, Guangzhou 510275*

^a^Current address: School of Materials Science and Engineering, South China University of Technology, Guangzhou 510641, P.R. China

*Corresponding author: *E-mail address*: lssljz@mail.sysu.edu.cn (J. Z. Liu)

Figure S The transcription levels of the *aroB*, *aroG^fbr^* and *tktA* genes in CIChE strains
